# Long term at-home treatment with transcranial direct current stimulation (tDCS) improves symptoms of cerebellar ataxia: a case report

**DOI:** 10.1186/s12984-019-0514-z

**Published:** 2019-03-19

**Authors:** Giuseppina Pilloni, Michael Shaw, Charles Feinberg, Ashley Clayton, Maria Palmeri, Abhishek Datta, Leigh E. Charvet

**Affiliations:** 1 Department of Neurology, New York University Langone Health, 10th floor, 222 East 41st Street, New York, NY 10017 USA; 2 Department of Mechanical Chemical and Materials Engineering, University of Cagliari, Cagliari, Italy; 3 Soterix Medical, New York, NY USA

**Keywords:** Cerebellar ataxia, Transcranial direct current stimulation (tDCS), Cerebellar tDCS, Remotely supervised tDCS, Telerehabilitation

## Abstract

**Background:**

Progressive cerebellar ataxia is a neurodegenerative disorder without effective treatment options that seriously hinders quality of life. Previously, transcranial direct current stimulation (tDCS) has been demonstrated to benefit cerebellar functions (including improved motor control, learning and emotional processing) in healthy individuals and patients with neurological disorders. While tDCS is an emerging therapy, multiple daily sessions are needed for optimal clinical benefit. This case study tests the symptomatic benefit of remotely supervised tDCS (RS-tDCS) for a patient with cerebellar ataxia.

**Methods:**

We report a case of a 71-year-old female patient with progressive cerebellar ataxia, who presented with unsteady gait and balance impairment, treated with tDCS. tDCS was administered using our RS-tDCS protocol and was completed daily in the patient’s home (Monday – Friday) with the help of a trained study technician. tDCS was paired with 20 min of simultaneous cognitive training, followed by 20 min of physical exercises directed by a physical therapist. Stimulation consisted of 20 min of 2.5 mA direct current targeting the cerebellum via an anodal electrode and a cathodal electrode placed over the right shoulder. The patient completed baseline and treatment end visits with neurological, cognitive, and motor (Lafayette Grooved Pegboard Test, 25 ft walk test and Timed Up and Go Test) assessments.

**Results:**

The patient successfully completed sixty tDCS sessions, 59 of which were administered remotely at the patient’s home with the use of real time supervision as enabled by video conferencing. Mild improvement was observed in the patient’s gait with a 7% improvement in walking speed, which she completed without a walking-aid at treatment end, which was in stark contrast to her baseline assessment. Improvements were also achieved in manual dexterity, with an increase in pegboard scores bilaterally compared to baseline.

**Conclusions:**

Results from this case report suggest that consecutively administered tDCS treatments paired with cognitive and physical exercise hold promise for improving balance, gait, and manual dexterity in patients with progressive ataxia. Remotely supervised tDCS provides home access to enable the administration over an extended period. Further controlled study in a large group of those with cerebellar ataxia is needed to replicate these findings.

**Trial registration:**

ClinicalTrials.gov Identifier: NCT03049969. Registered 10 February 2017- Retrospectively registered.

## Background

Progressive cerebellar ataxias are the result of diverse disease processes that can be genetic or acquired [1, 2]. Cerebellar ataxias are characterized clinically by oculomotor deficits, dysarthria, limb dysmetria, delay in movement initiation, dyskinesia, and kinetic tremor [3]. Among the wide spectrum of motor signs, ataxic gait is the most relevant and it is characterized by unsteadiness, increased step width, reduced step length, slow walking speed, variable foot placement and irregular foot trajectories [1, 4]. Such unsteady movements and variable gait patterns may be caused by deficits either in dynamic inter- and intra-limb coordination or in balance control [1]. During the clinical course of the disease, patients with cerebellar dysfunction may endure slowed reaction times, or limitations in cognitive domains, such as attention, memory and flexibility [5]. Taken together, the symptom burden can negatively impact mood, productivity, and quality of life in patients.

Currently, therapeutic approaches for cerebellar disorders rely heavily on rehabilitation since there are no pharmacological evidence-based treatments [6], which has led to interest in finding innovative techniques to improve clinical symptoms in this wide spectrum of debilitating disorders [6, 7]. Transcranial direct current stimulation (tDCS), a non-invasive brain stimulation technique, has demonstrated beneficial effects in modulating several cerebellar skills, including motor control and learning and emotional processing in both healthy patients as well as those with neurological disorders [8, 9]. tDCS is presumed to increase cortical excitability and is often administered simultaneously with another intervention to achieve clinical benefit [10]. Based on this concept, tDCS is widely considered as a complementary technique in conjunction with motor and/or cognitive rehabilitation [11, 12].

Studies have highlighted the therapeutic potential of cerebellar tDCS in modulating behavioral performance and reducing motor and neurocognitive symptoms for those with cerebellar ataxia [7, 8, 13–16]. Additionally, other studies have shown patients with progressive cerebellar ataxia to attain positive clinical outcomes in upper limb tremor, dysmetria, gait, postural control, and finger dexterity after only one session of cerebellar tDCS [15, 17]. The effects of tDCS have been demonstrated to persist beyond the acute stimulation period, and, additionally, repeated and consecutive tDCS treatments have been found to produce longer persisting changes in brain excitability [17] and clinically relevant effects [10, 15]. After ten tDCS sessions targeting the cerebellum and spine, a reduction in motor symptoms and improvement in quality of life in patients with neurodegenerative ataxia was reported [13]. Most recently cerebellar tDCS has been shown to lead to improved outcomes in patients with clinical ataxia in a two-week controlled trial [14]. Evidence from these clinical studies that investigated the role of tDCS in modulating the activity of the cerebellum in ataxia disorders reported changes in walking patterns, posture control, and motor learning [14, 18]. In sum, despite few publications testing the effects of multiple cerebellar tDCS sessions in patients with cerebellar ataxia, all have reported significant and lasting improvement in ataxic symptoms and physiological cerebellar brain inhibition pathways [13, 14, 17, 18].

As cumulative treatment sessions appear to lead to the strongest clinical benefit, extended treatment is needed in order to enhance the outcome of rehabilitation for individuals with cerebellar ataxia. Unfortunately, real-world obstacles have prevented completion of extended treatment schedules in clinical trials as the burden of time and travel on the patient to receive daily sessions in the clinic is great. As a solution, we have developed and extensively validated a remotely supervised or RS-tDCS protocol for patients to self-administer tDCS in their homes while being monitored in real-time via video conferencing [11, 19–28]. This provides strict clinical supervision while enabling study protocols with a greater number of stimulation sessions than previously reported [11, 21, 29].

Here we report a case of real-world clinical tDCS application, where a patient with cerebellar ataxia completed extended tDCS treatments from home. Following previous studies demonstrating the beneficial effects of tDCS for those with cerebellar ataxia, the purpose of this study was to document and assess how an extended schedule of daily tDCS sessions targeting the cerebellar pathway would improve the symptoms of progressive cerebellar ataxia.

## Case report

The patient was a 71-year-old female with a history of progressive cerebellar ataxia. She first experienced the onset of her current illness nine years ago, when she developed unsteady gait with difficulty performing a tandem walk.

She was initially treated for suspected inner ear problems, but approximately two years ago, magnetic resonance imaging (MRI) revealed cerebellar atrophy, which has led to her current diagnosis. Neuro-ophthalmologic evaluation indicated ophthalmoplegia, requiring her to wear prisms lenses to assist with reading.

To date, her symptoms have gradually progressed with notable worsening over the past year, and include balance disorder, increased risk of falling, reduced manual dexterity, fatigue, and episodes of speech slurring. She uses a cane for ambulation with difficulty turning and moving between a standing to seated position. She tried several pharmacological treatments over the years without any lasting clinical benefits. She completes daily-prescribed physical rehabilitation training exercises at home.

## Methods

Multiple sessions of consecutively delivered tDCS are described as having a greater potential for efficacy than treatments that are infrequent or are temporally distant [11, 21, 25]. To enable remote delivery of an extended tDCS treatment schedule to this patient, she was enrolled in an open-label  exploratory tDCS protocol. The patient provided written informed consent to receive this remotely supervised tDCS treatment. All study procedures were approved by the NYU Langone Health Institutional Review Board.

The procedure for training and at home self-administration followed the RS-tDCS protocol [19, 20, 22–26]. At the baseline visit, after training with a technician, tolerability and capacity for self-administration were determined and the participant completed her first tDCS session in clinic as part of training procedures. This session was followed by 59 remotely supervised sessions using a HIPAA compliant video conferencing platform. tDCS sessions were completed daily, in the morning, 5 days a week for eight weeks. After the 40th session, the participant took a two-week break before completing another 20 sessions.

**Fig. 1:**
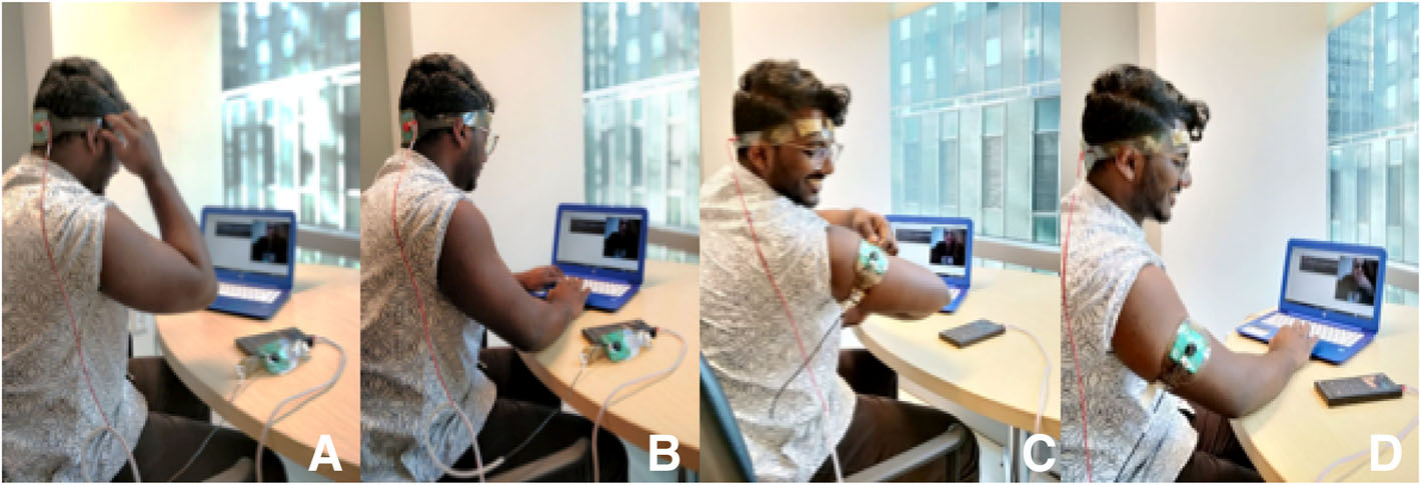
Example of the RS-tDCS kit and the electrodes preparation and positioning: tDCS headstrap for electrode cerebellar montage with the anode aligned with the median line over the cerebellum and the cathode over the right shoulder; stimulation device; single-use pre-saturated electrodes; laptop. **a** and **b** showed the positioning of the headstrap and the checking of its correct placement by the study technician connected via video conferencing. **c** and **d** showed the positioning of the cathode over the right shoulder and the releasing of the code to unlock the stimulation device for starting the session

**Fig. 2:**
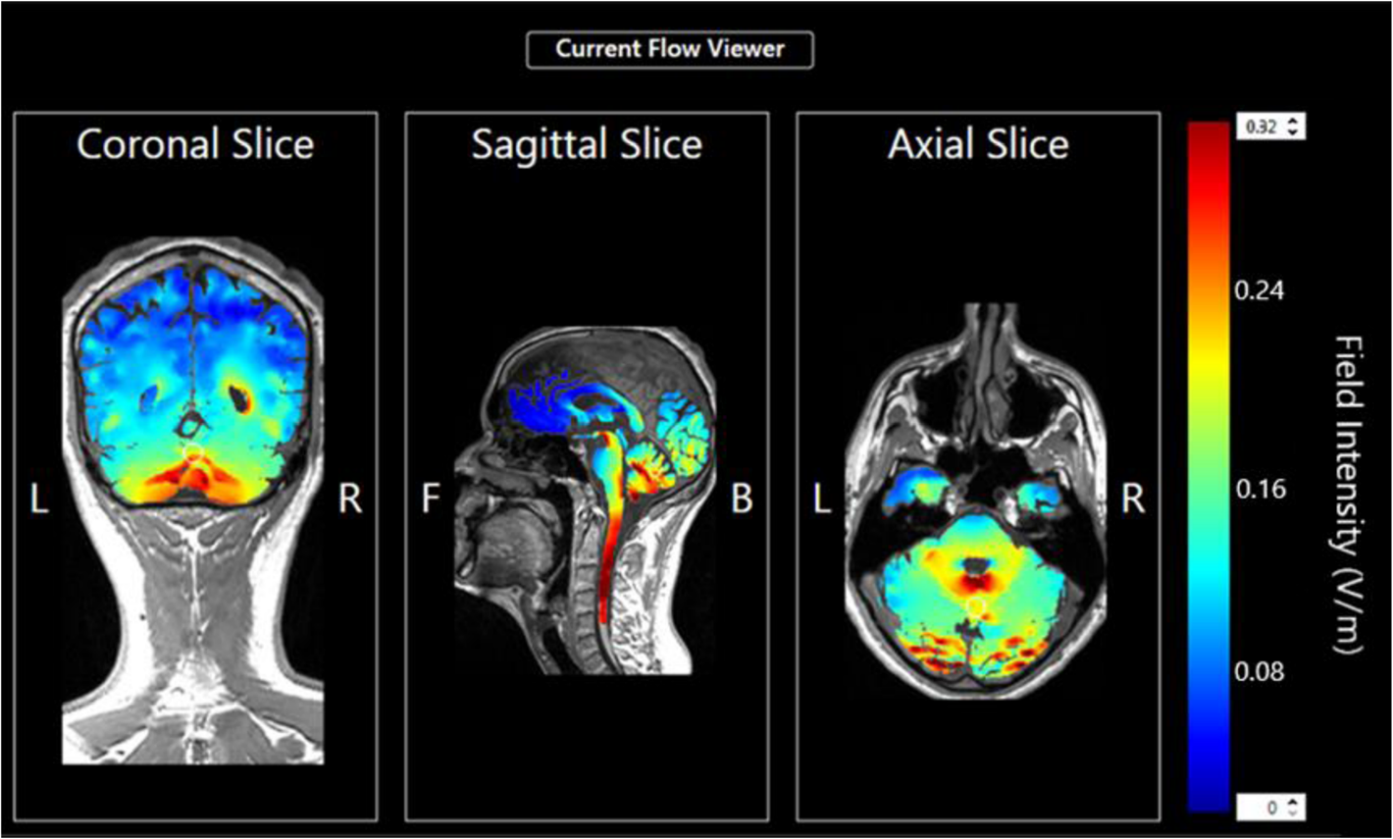
Modelling of the electric field intensity of cerebellar electrode placement. Theoretical model of electric field distribution generates using anodal electrode on the medial line over the whole cerebellum and cathodal electrode on the right shoulder at 2.5 mA. The stimulation montage targeting the cerebellar region is tailored by generating the current flow using the HD-Explore software (Soterix Medical, NY, USA)

**Fig. 3:**
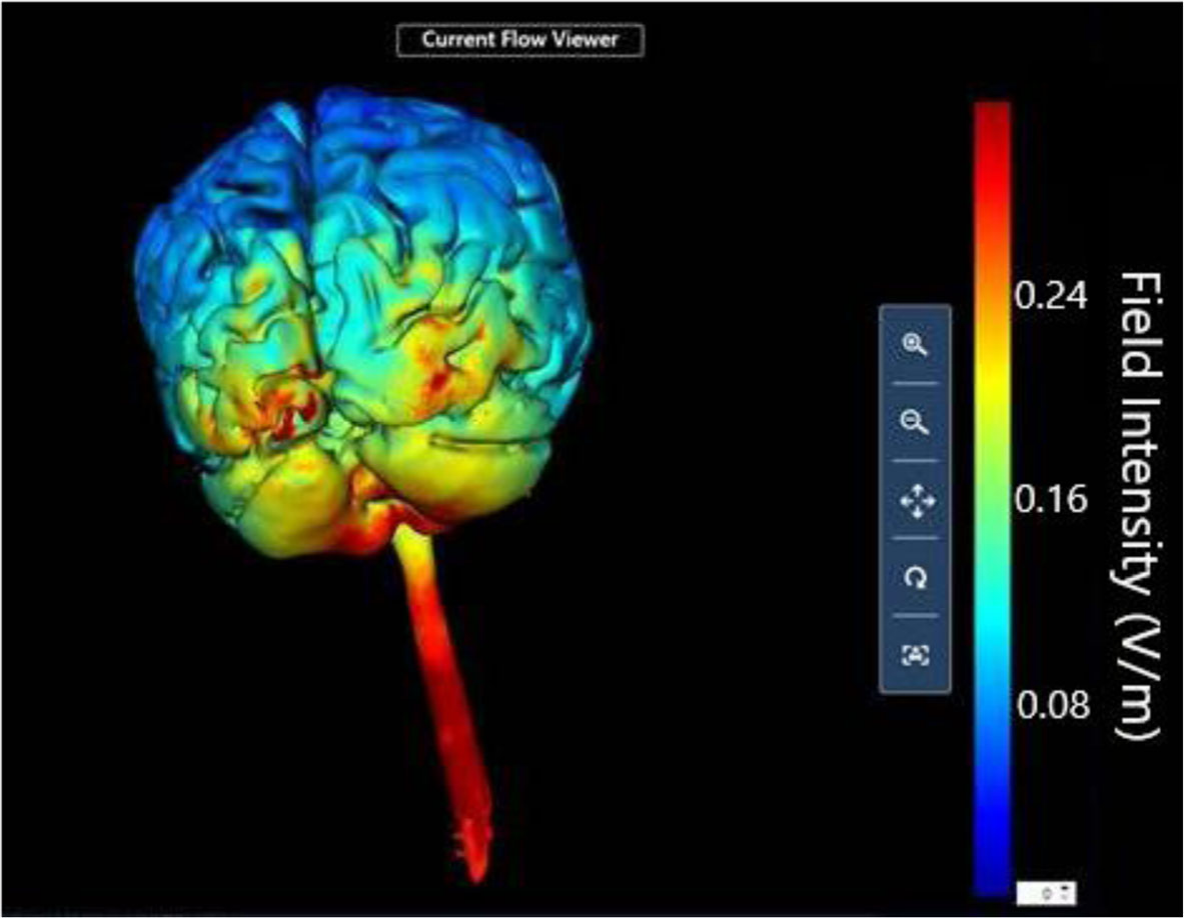
3-D Model showing the electric field intensity distribution of cerebellar electrode placement. 3-D theoretical model of electric field distribution generates using anodal electrode over the cerebellum and cathodal electrode on the right shoulder at 2.5 mA. The current flow distribution is generated using the HD-Explore software (Soterix Medical, NY, USA)

tDCS was delivered utilizing a Soterix Medical mini-CT device that operates by a single use “unlock” code, provided to the participant in advance of each daily session after meeting safety clearance and headset placement. Each session consisted of 20 min, 2.5 mA continuous direct current, applied by saline-soaked surface sponges (surface 25 cm^2^) attached to a customized headstrap with electrodes targeting the cerebellar region. The anodal electrode was placed on the median line over the cerebellum, while the cathodal electrode was placed on the right shoulder (Fig. 1) [30]. The theoretical distribution of the electric field intensity in the cerebellar electrode montage is shown in Figs. 2 and 3 [31].

Per the RS-tDCS protocol [11, 19, 21, 22, 25–27, 32], computer-based cognitive training exercises targeting processing speed, attention and working memory were performed simultaneously with the stimulation [33]. The cognitive training consisted of a pre-selected assortment of computerized cognitive games based on five traditional tasks: n-back, auditory and visual span, simple arithmetic, and match-to-sample. Immediately after each session, the participant completed 20 min of physical exercise as prescribed by her physical therapist for improving postural stability, reinforcing physical endurance and core strengthening [34]. It was a standard routine repeated each day for practice (Table 1).

**Table 1:** Physical Exercise Program

Physical Domain	Exercise	Repetitions
Kinesthetic warm up	• Side step • Forward step • Backward step • Front and back cross-step	30 repetitions each leg
Core strengthening	• Standing anterior-posterior weight shift • Rise from chair with arms crossed	20 repetitions
Static and dynamic balance	• Standing position with legs apart and arms crossed • Standing position with feet together	10 repetitions open/close eyes x 30s
	• Standing position up on toe • Standing position up on heels	10 repetitions open eyes x 30s
	• Turning in circle	3 repetitions in both directions

The protocol included a baseline visit consisting of a neurological assessment, cognitive testing, and administration of motor tests. Motor assessments were repeated after both the 40th and 60th sessions, and cognitive and neurological assessments were conducted again after the 60th session during her follow up visit.

### Evaluation procedures

#### Motor assessment

Fine motor function was measured with the Lafayette Grooved Pegboard Test [35], administered separately for each hand to evaluate manual dexterity and upper limb coordination. The pegboard has 25 grooved holes arranged in rows of five; the shape of each hole is identical, but the orientation varies so that subject must rotate the peg to match the hole before it can be inserted. The patient was instructed to put 25 pegs in the holes in a fixed order from side to side and from top to bottom, as quickly as possible. The recorded score was the total time in seconds to complete a trial, for each separate hand (dominant and non-dominant). Adjusted age-normative z scores were computed for both hands [36].

Gait was assessed with the 25 ft walking test (25FWT), defined as the time needed to walk 25 ft, as quickly as possible but safely, with the assistance of any walking-aid if needed.

The Timed Up and Go Test (TUG) was used to assess functional mobility. The TUG test measures the patient’s ability to stand up from a chair with armrests, walk 3 m, turn, walk back and sit down. Assistance is not permitted, but a walking-aid is allowed. The time needed to perform this task is the TUG test score.

All motor assessments used in this case study have been shown to be reliable and accurate and are commonly used to assess motor functions in neurological disease.

#### Clinical assessment

The participant completed self-reported questionnaires measuring fatigue, the Fatigue Severity Scale (FSS) [37], and Patient-Reported Outcomes Measurement Information System (PROMIS) – Fatigue [38]. The FSS consists of nine items assessing frequency and severity of fatigue in the last past week, with higher scores indicative of more severe fatigue. The PROMIS- Fatigue assesses the impact and experience of fatigue during the past week. Item responses are rated on a five-point scale ranging from “never” to “always”. Higher scores are indicative of more fatigue.

Mood and affect were measured by the Positive and Negative Affect Schedule (PANAS) [39] and the PROMIS- Positive Affect (PA) scale. Positive affect reflects the extent to which a person feels enthusiastic, active and alert, while negative affect is a general dimension of subjective distress and unpleasant engagement. The PANAS is a twenty-item measure reflecting the extent of positive and negative affect felt over the past week. Item responses are rated on a five-point scale, with the total score separated into both a positive and negative affect score, ranging from 10 to 50, where higher scores representing higher levels of positive/negative affect. The PROMIS-PA scale is a nine-item measure reflecting the extent to which a person feels enthusiastic, active and alert. Item responses are rated on a five-point scale from “never” to “always”. Information about daytime sleepiness, pain and tDCS tolerability were recorded for each stimulation session as well.

## Results

Following RS-tDCS protocol guidelines [11, 21–23, 25–27], the patient demonstrated competence and aptitude to complete her treatments at home and learned how to self-administer treatment at the first treatment session. Including the initial in-clinic tDCS session, the training procedures took approximately one hour at the baseline treatment session to complete. Then, using the remote supervision procedures with video conferencing, she successfully completed the following 59 sessions from her home. Treatment was well-tolerated, and she did not report any treatment-limiting adverse events related to the tDCS treatment.

Mild to moderate improvement was observed across multiple domains for this patient following treatment as compared with her baseline performance (see Table 2). Interestingly, the patient was able to complete the post-treatment motor assessments without a walking-aid, whereas she relied on a cane to stabilize herself at baseline. A mild improvement was observed in the 25-FWT, with the patient completing the test 7% faster. The patient improved on the TUG Test, with a post-treatment completion time of 9.88 s compared to 11.90 s at baseline. Noticeable improvements were also achieved in manual dexterity, with pegboard scores improving bilaterally from the baseline assessment. The patient performed 18% faster with the dominant hand, and 19% faster with the non-dominant hand, with a reduction amount to 2.07 and 1.92 in the z-score for the dominant and non-dominant hand, respectively.

**Table 2:** Motor assessment main parameters

Motor Assessment				
Motor Functioning Measure		Baseline	40th	60th
T25-FW [s]		6.96 ± 0.13^a^	7.37 ± 0.12	6.48 ± 0.07
TUG [s]		11.90 ± 0.52^a^	9.88 ± 0.06	12.35 ± 0.19
PEGS [s]	Dominant	205.00	172.00	168.14
	Non- Dominant	260.00	181.00	209.95
PEG z-score	Dominant	−6.54	−4.77	− 4.57
	Non- Dominant	−6.57	−3.55	−4.65

The symbol ^a^ indicate the use of walking aid (cane) for the performance of the test

The patient reported a reduction in perceived fatigue, from 22 to 14 on the FSS and from 14 to 10 on the PROMIS-Fatigue (See Table 3). At baseline, cognitive functioning was intact across all measures without indication of clinical impairment. Findings from the clinical measures repeated at the follow up visit did not indicate any notable change in the areas assessed.

**Table 3:** Clinical assessment main parameters

Clinical Assessment		
Clinical Measures	Baseline	60th
FSS	22	**14**
PROMIS - Fatigue	14	**10**
PROMIS – Positive Affect	43	**45**
PANAS - SF Positive Affect	38	**44**
PANAS – SF Negative Affect	10	10

The scores reported in bold means an improvement at 60th session

## Discussion

This report describes the benefit and feasibility of an extended schedule of RS-tDCS paired with cognitive and motor rehabilitation for a patient with cerebellar ataxia. The patient had improvement in functional motor aspects, balance capacity, fatigue, and positive affect. Full treatment compliance was observed on the part of the patient.

We have now demonstrated the feasibility of our RS-tDCS protocol to deliver at-home tDCS under clinical supervision in participants with a range of neurologic disabilities. We have established methods and completed preliminary trials in participants with MS [11, 21–23, 27], and, more recently, in Parkinson’s disease [25, 26]. Across these studies, participants of all ages (18 to 75 years) and levels of disability (including those wheelchair- and/or caregiver-dependent) have been successful in receiving tDCS at home. The RS-tDCS protocol maintains the standards of clinic administration, while allowing for the extended protocols that we believe are necessary to have optimal and continued benefit. This case study provides preliminary support for the use of the RS-tDCS protocol for the clinical population of cerebellar ataxia.

Furthermore, this case study supports the clinical role of tDCS and cognitive and motor rehabilitation to improve balance and ambulatory abilities for individuals with cerebellar ataxia, as shown by improved performance on the 25-FW and TUG test from baseline to follow up visit following treatment. The patient was dependent on her walking aid at the first assessment, but was able to complete the motor measures independently after treatment. Interestingly, consistent with previous reports of extended treatment [12, 13], she experienced a cumulative benefit across the sixty tDCS sessions. The patient also reported experiencing direct benefit, noticing a longer endurance for standing in general and a direct improvement in static upright posture and base of support.

Regarding the movement of upper limbs, we observed a large improvement in fine manual dexterity, as shown by a reduction in time performing the pegboard test, bilaterally. Subjectively, the participant reported that she experienced an enhancement of movement quality from about the first thirty sessions onward. She also reported, in particular, an improvement in manual activities and in attending daily activities that required a deal of standing and walking.

Consistent with the results of previous studies in multiple sclerosis (using a different montage) [40, 41], tDCS was effective in improving perceived fatigue, as evidenced by change in fatigue scores. This is consistent with the patient’s own improvement in self-reported fatigue.

Recent studies have investigated the potential of tDCS of the cerebellum in regulating synaptic plasticity in motor cortical networks, also providing evidence that the cerebellum contributes to the learning processes underlying motor adaptation both in healthy and degenerative cerebellar diseases. Further support for the potential therapeutic efficacy of cerebellar tDCS strategies in the neurorehabilitation of ataxic gait has recently been provided by evidence that anodal tDCS applied over the cerebellum can induce significant clinical improvement in patients with neurodegenerative ataxia, also providing evidence that long-term gain can be made with a protocol involving multiple stimulation sessions [9]. The results of this case study are in part supported by the interesting therapeutic effect observed by Benussi and colleagues that reported significant clinical motor improvement in ataxic patients [14, 15].

In their first double-blind, randomized, sham-controlled study, Benussi and colleagues [15] showed functional but temporary improvement in gait and hand dexterity in patients with ataxia after a single session of cerebellar tDCS. Additionally, in their more recent study involving 10 sessions over two weeks, they report evidence of long-lasting motor effects in an 8-m walking test and in the 9-hole peg test [13, 14]. Evidence further supporting the observed clinical improvement is related to an increase in excitability of the cerebellar motor-cortex, as demonstrated by an increase of the cerebellar brain inhibition [13, 14]. Some authors showed also that one session of anodal tDCS applied to the right cerebellar hemisphere reduced postural tremor and amplitude of the oscillation in ataxia, with slight improvement in dysmetria [17, 18].

The improvements, achieved by means of tDCS, may reflect more effective cerebellar control over motor function, supporting the current hypotheses that anodal cerebellar tDCS restores the inhibitory effect exerted by Purkinje neurons upon cerebellar nuclei, promoting appropriate patterns of nuclear discharge [16]. This inhibitory effect of cerebellar nuclei would improve motor aspects. In our patient, cerebellar tDCS reduced unsteadiness of the walking and finger dexterity.

Results from this case report suggest that multiple RS-tDCS sessions are promising for improving balance, gait and manual dexterity in patients with progressive ataxia. Better functional gains in walking and finger dexterity, however, may be achieved with the simultaneous practice of tDCS and physical exercise. This suggests that, in addition to intensity, targeting the area of the cerebellum and number of sessions performed are critical factors in determining outcomes [30]. Findings from this case report suggest that offline effects (post-stimulation) of tDCS are effective in enhancing the outcome of physical protocols performed after the stimulation session. Targeted studies are needed to define various issues concerning the application of tDCS for therapeutic purposes in cerebellar ataxia, e.g. which areas are the most beneficial for stimulation, when patients should perform physical exercise, and even which clinical features should be considered in individual patients, to guide the choice of the best stimulation parameters.

There are several limitations to this case study. As the treatment was open-label, there is no way to determine the role of any potential for a placebo effect in the observed benefits of the treatment. Additionally, this study lacked specific measures to assess the ataxic symptoms of our participant, and specifically, the Scale for Assessment and Rating of Ataxia (SARA) [42], thereby limiting the interpretability of this case study’s results. Following the standard RS-tDCS protocol, computer-based cognitive training games were completed during the stimulation period. In addition to potential cognitive remediation, this serves the purpose of having a uniform activity across all sessions (and, in larger studies, across participants), and also has the participant remain seated and observable by the supervising study team member. However, greater clinical benefit may have been achieved if the active stimulation period was paired with physical rehabilitation or exercise, which may produce stronger clinical effects. Finally, the generalizability of case studies can be limited, especially considering our study, which worked with a single patient.

While these findings are promising for the extended administration of RS-tDCS and the treatment of motor symptoms and fatigue in cerebellar ataxia, larger and controlled trials are needed to guide clinical use.

## Conclusion

The case study supports feasibility of the remotely supervised tDCS protocol for use with ataxic populations. Since there is currently no approved therapy to treat cerebellar motor dysfunction, based on the results of this case study, multiple tDCS treatments targeting the cerebellum should be considered a promising neurorehabilitation tool for improving motor symptoms in patients with cerebellar ataxia.

## Abbreviations

25FWT: 25 ft walking test
FSS: Fatigue Scale Severity
PANAS-SF: Positive and Negative Aspect Scales
PEGS: Lafayette Grooved Pegboard Tests
PROMIS: Patient-Reported Outcomes Measurement Information System
RS-tDCS: Remotely Supervised Transcranial Direct Current Stimulation
SARA: Scale for Assessment and Rating of Ataxia
tDCS: Transcranial Direct Current Stimulation
TUG: Time Up and Go

## References

[1] Ilg W, Golla H, Thier P, Giese MA (2007). Specific influences of cerebellar dysfunctions on gait. Brain..

[2] Stoodley CJ (2012). The cerebellum and cognition: evidence from functional imaging studies. Cerebellum..

[3] Diener HC, Dichgans J (1992). Pathophysiology of cerebellar ataxia. Mov Disord.

[4] Morton SM, Bastian AJ (2007). Mechanisms of cerebellar gait ataxia. Cerebellum..

[5] Pope PA, Miall RC (2014). Restoring cognitive functions using non-invasive brain stimulation techniques in patients with cerebellar disorders. Front Psychiatry.

[6] Zesiewicz TA, Wilmot G, Kuo SH, Perlman S, Greenstein PE, Ying SH (2018). Comprehensive systematic review summary: treatment of cerebellar motor dysfunction and ataxia: report of the guideline development, dissemination, and implementation Subcommittee of the American Academy of neurology. Neurology..

[7] Fujioka S, Tsuboi Y, Friedman JH (2018). A novel promising therapeutic approach for patients with ataxic disorders?. Neurology..

[8] Franca C, de Andrade DC, Teixeira MJ, Galhardoni R, Silva V, Barbosa ER (2018). Effects of cerebellar neuromodulation in movement disorders: a systematic review. Brain Stimul..

[9] Ferrucci R, Priori A (2014). Transcranial cerebellar direct current stimulation (tcDCS): motor control, cognition, learning and emotions. Neuroimage..

[10] Brunoni AR, Nitsche MA, Bolognini N, Bikson M, Wagner T, Merabet L (2012). Clinical research with transcranial direct current stimulation (tDCS): challenges and future directions. Brain Stimul.

[11] Charvet L, Shaw M, Dobbs B, Frontario A, Sherman K, Bikson M (2018). Remotely supervised transcranial direct current stimulation increases the benefit of at-home cognitive training in multiple sclerosis. Neuromodulation..

[12] Sanchez-Kuhn A, Perez-Fernandez C, Canovas R, Flores P, Sanchez-Santed F (2017). Transcranial direct current stimulation as a motor neurorehabilitation tool: an empirical review. Biomed Eng Online.

[13] Benussi A, Dell'Era V, Cantoni V, Bonetta E, Grasso R, Manenti R (2018). Cerebello-spinal tDCS in ataxia: a randomized, double-blind, sham-controlled, crossover trial. Neurology..

[14] Benussi A, Dell'Era V, Cotelli MS, Turla M, Casali C, Padovani A (2017). Long term clinical and neurophysiological effects of cerebellar transcranial direct current stimulation in patients with neurodegenerative ataxia. Brain Stimul..

[15] Benussi A, Koch G, Cotelli M, Padovani A, Borroni B (2015). Cerebellar transcranial direct current stimulation in patients with ataxia: a double-blind, randomized, sham-controlled study. Mov Disord.

[16] Grimaldi G, Argyropoulos GP, Bastian A, Cortes M, Davis NJ, Edwards DJ (2016). Cerebellar transcranial direct current stimulation (ctDCS): a novel approach to understanding cerebellar function in health and disease. Neuroscientist..

[17] Bodranghien F, Oulad Ben Taib N, Van Maldergem L, Manto M (2017). A postural tremor highly responsive to transcranial Cerebello-cerebral DCS in ARCA3. Front Neurol.

[18] Grimaldi G, Oulad Ben Taib N, Manto M, Bodranghien F (2014). Marked reduction of cerebellar deficits in upper limbs following transcranial cerebello-cerebral DC stimulation: tremor reduction and re-programming of the timing of antagonist commands. Front Syst Neurosci.

[19] Charvet L, Kasschau M, Datta A, Knotkova H, Stevens M, Alonzo A (2015). Remotely-supervised transcranial direct current stimulation (tDCS) for clinical trials: guidelines for technology and protocols. Front Syst Neurosci.

[20] Shaw MT, Kasschau M, Dobbs B, Pawlak N, Pau W, Sherman K, et al. Remotely supervised transcranial direct current stimulation: an update on safety and tolerability. J Vis Exp. 2017;128. https://www.jove.com/video/56211/remotelysupervised-transcranial-direct-current-stimulation-an-update. 10.3791/56211 PMC5752383 29053684

[21] Charvet L, Dobbs B, Shaw M, Bikson M, Datta A, Krupp LB. Remotely supervised transcranial direct current stimulation for the treatment of fatigue in multiple sclerosis: results from a randomized, sham-controlled trial. Mult Scler J. 2017;24(13):1760–69. 10.1177/1352458517732842 PMC5975187 28937310

[22] Kasschau M, Reisner J, Sherman K, Bikson M, Datta A, Charvet L (2016). Transcranial direct current stimulation is feasible for remotely supervised home delivery in multiple sclerosis. Neuromod..

[23] Kasschau M, Sherman K, Haider L, Frontario A, Shaw M, Datta A (2015). A protocol for the use of remotely-supervised transcranial direct current stimulation (tDCS) in multiple sclerosis (MS). Jove-J Vis Exp.

[24] Alonzo A, Charvet L. Home-based tDCS: design, feasibility and safety considerations. In: André Brunoni, Michael Nitsche, Colleen Loo, editors. Transcranial direct current stimulation in neuropsychiatric disorders. Springer; 2016. p. 351–61.

[25] Dobbs B, Pawlak N, Biagioni M, Agarwal S, Shaw M, Pilloni G (2018). Generalizing remotely supervised transcranial direct current stimulation (tDCS): feasibility and benefit in Parkinson’s disease. J. Neuroeng. Rehabil.

[26] Agarwal S, Pawlak N, Cucca A, Sharma K, Dobbs B, Shaw M (2018). Remotely-supervised transcranial direct current stimulation paired with cognitive training in Parkinson’s disease: an open-label study. J Clin Neurosci.

[27] Clayton AM, Howard J, Dobbs B, Shaw MT, Charvet LE (2018). Remotely supervised transcranial direct current stimulation after ECT improves mood and cognition in a patient with multiple sclerosis: a case study. J ECT.

[28] Bikson M, Brunoni AR, Charvet LE, Clark VP, Cohen LG, Deng Z-D (2018). Rigor and reproducibility in research with transcranial electrical stimulation: an NIMH-sponsored workshop. Brain stimulation.

[29] Dobbs B, Pawlak N, Shaw MT, Clayton A, Sherman K, Charvet LE. Remotely-Supervised Transcranial Direct Current Stimulation (RS-tDCS) is Feasible for 40 Treatment Sessions (P5.005). Neurology. 2018;90(Suppl 15): P5.005.

[30] Ferrucci R, Cortese F, Priori A (2015). Cerebellar tDCS: how to do it. Cerebellum..

[31] Datta A, Bansal V, Diaz J, Patel J, Reato D, Bikson M (2009). Gyri-precise head model of transcranial direct current stimulation: improved spatial focality using a ring electrode versus conventional rectangular pad. Brain Stimul..

[32] Charvet L, Shaw M, Dobbs B, Frontario A, Sherman K, Bikson M, et al. Remotely supervised transcranial direct current stimulation increases the benefit of at-home cognitive training in multiple sclerosis. Neuromod. 2017. 10.1111/ner.12583 PMC5975186 28225155

[33] CogState 2015 [cited 2015 February 10, 2015]. Available from: http://cogstate.com/

[34] Keller JL, Bastian AJ (2014). A home balance exercise program improves walking in people with cerebellar ataxia. Neurorehabil Neural Repair.

[35] Matthews C, Klove H (1964). Instruction manual for the adult neuropsychology test battery.

[36] Heaton RK, Grant I, Matthews CG (1986). Differences in neuropsychological test performance associated with age, education, and sex. Neuropsychological assessment of neuropsychiatric disorders.

[37] Krupp LB, LaRocca NG, Muir-Nash J, Steinberg AD (1989). The fatigue severity scale. Application to patients with multiple sclerosis and systemic lupus erythematosus. Arch Neurol.

[38] Jensen RE, Moinpour CM, Potosky AL, Lobo T, Hahn EA, Hays RD (2017). Responsiveness of 8 patient-reported outcomes measurement information system (PROMIS) measures in a large, community-based cancer study cohort. Cancer..

[39] Kay SR, Fiszbein A, Opler LA (1987). The positive and negative syndrome scale (PANSS) for schizophrenia. Schizophr Bull.

[40] Charvet LE, Dobbs B, Shaw MT, Bikson M, Datta A, Krupp LB. Remotely supervised transcranial direct current stimulation for the treatment of fatigue in multiple sclerosis: results from a randomized, sham-controlled trial. Mult Scler 2017:1352458517732842. 10.1177/1352458517732842 PMC5975187 28937310

[41] Ferrucci R, Vergari M, Cogiamanian F, Bocci T, Ciocca M, Tomasini E (2014). Transcranial direct current stimulation (tDCS) for fatigue in multiple sclerosis. NeuroRehabilitation..

[42] Schmitz-Hübsch T, Du Montcel ST, Baliko L, Berciano J, Boesch S, Depondt C (2006). Scale for the assessment and rating of ataxia: development of a new clinical scale. Neurology..

